# Mitofusin2 expression is associated with podocyte injury in IgA nephropathy

**DOI:** 10.1186/s40001-023-01107-5

**Published:** 2023-03-30

**Authors:** Xuanli Tang, Yuan Yuan, Xiaoli Liang, Xue Jiang

**Affiliations:** Department of Nephrology (Key Laboratory of Zhejiang Province, Management of Kidney Disease), Hangzhou TCM Hospital Affiliated to Zhejiang Chinese Medical University, Hangzhou, 310007 China

**Keywords:** IgA nephropathy, Mitofusin2, Podocyte

## Abstract

**Background:**

Podocyte injury is associated with IgA nephropathy (IgAN) prognosis. Mitochondrial dysfunction is a major contributor to podocyte injury and death. Mitofusin2 (Mfn2) plays an important role in regulating the morphology and function of mitochondria. This study aimed to investigate the potential of Mfn2 as a biomarker to evaluate the degree of podocyte injury.

**Methods:**

This single-center, retrospective study enrolled 114 patients with biopsy-proven IgAN. Immunofluorescence and TUNEL staining were applied, and clinical and pathological features were compared between patients with different patterns of Mfn2 expression.

**Results:**

In IgAN, Mfn2 is mainly expressed in podocytes and significantly associated with nephrin, TUNEL, and Parkin staining. Among the 114 IgAN patients, 28 (24.56%) did not exhibit Mfn2 expression in podocytes. The patients in the Mfn2-negative group had lower serum albumin (34.43 ± 4.64 g/L vs. 36.48 ± 3 .52 g/L, *P* = 0.015) and estimated glomerular filtration rate (eGFR) (76.59 ± 35.38 mL/min vs. 92.13 ± 25.35 mL/min, *P* = 0.013), higher 24 h proteinuria (2.48 ± 2.72 g/d vs. 1.27 ± 1.31 g/d, P = 0.002), serum creatinine (Scr) (107.39 ± 57.97 μmol/L vs. 84.70 ± 34.95 μmol/L, *P* = 0.015), blood urea nitrogen (BUN) (7.36 ± 4.45 mmol/L vs. 5.68 ± 2.14 mmol/L, *P* = 0.008), and higher S/T scores (92.86% vs. 70.93% and 42.85% vs. 15.12%, respectively, *P* < 0.05).

In the Mfn2-negative group, the mitochondria were punctate and round ridges disappeared, and a lower length-to-width ratio and much higher M/A ratio were observed. Correlation analysis showed that the intensity of Mfn2 was negatively correlated with Scr (r = − 0.232, *P* = 0.013), 24 h proteinuria (r = − 0.541, *P* = 0.001), and the degree of podocyte effacement (r = − 0.323,* P* = 0.001), and positively correlated with eGFR (r = 0.213,* P* = 0.025).

Logistic regression analysis showed that the Mfn2-negative group had a higher risk of severe podocyte effacement (≥ 50%) (OR = 3.061,* P* = 0.019).

**Conclusion:**

Mfn2 was negatively correlated with proteinuria and renal function. A lack of Mfn2 in podocytes indicates severe podocyte injury and a high degree of podocyte effacement.

## Introduction

Immunoglobulin A (IgA) nephropathy (IgAN) is the most common primary glomerulonephritis in the world [1]. As the leading cause of end-stage renal disease (ESRD), approximately 10–60% of patients with IgAN progress to kidney failure within 10–20 years [1, 2]. Mesangial cell proliferation and IgA1-predominant mesangial deposits are pathological hallmarks of IgAN. Previous studies have suggested that podocyte injury also occurs in IgAN and is associated with the pathogenesis of Gd-IgA1. Podocyte injury is usually associated with significant proteinuria, manifested as foot process effacement, which is considered to be a key factor leading to progression and poor prognosis in IgAN [3–5].

Podocytes are terminally differentiated and have a poor proliferative capacity. Mitochondrial dysfunction is a major contributor to podocyte injury and death [6, 7]. Various mitochondrial dysfunction pathways have been identified as the main molecular mechanisms of podocyte injury, such as elevated mitochondrial ROS production [8], imbalanced mitochondrial dynamics [9], and decreased mitochondrial biogenesis [10, 11].

Mitofusin2 (Mfn2) was initially identified as a dynamin-like protein involved in fusion of the outer mitochondrial membrane (OMM) that participates in mitochondrial fusion and contributes to the maintenance of the mitochondrial network [12]. Moreover, Mfn2 is involved in the clearance of damaged mitochondria, serves as a mitochondrial receptor for Parkin (E3 ubiquitin ligase), and facilitates the recruitment of Parkin to the impaired mitochondria, which participates in mitophagy [13–15]. Our previous study showed that Mfn2 deficiency participates in podocyte injury in a focal segmental glomerulosclerosis (FSGS) animal model by inhibiting Pink1/Parkin-associated mitophagy. In diabetic kidney disease (DKD), Cao et al. [16] reported that Mfn2 regulates the morphology and functions of mitochondria-associated ER membranes (MAMs) and mitochondria by inhibiting the PERK pathway and exerts anti-apoptotic effects on podocytes. Whether Mfn2 participates in podocyte injury and is related to clinical and pathological characteristics in IgA nephropathy has not been reported to date. Therefore, this study aimed to explore the relationship between Mfn2 expression and podocyte injury and further elucidate its potential predictive value for IgAN prognosis.

## Methods

### Patients

Patients aged ≥ 18 years with biopsy-proven IgAN in the Hangzhou Hospital of Traditional Chinese Medicine between April 2022 and August 2022 were enrolled, and secondary causes of IgAN, such as liver or inflammatory bowel diseases, other autoimmune disorders, infections, and Henoch–Schönlein purpura, were excluded. Clinical data, including sex, age, proteinuria, serum creatinine (Scr), blood urea nitrogen (BUN), serum albumin (ALB), blood pressure, and serum IgA, were collected at the time of biopsy.

### Histopathology

Renal histological lesions were graded based on the MEST-C score [17]. M0/M1 was defined as ≤ / > 50% of glomeruli exhibiting mesangial hypercellularity, E0/E1 as the absence/presence of endocapillary hypercellularity, S0/S1 as the absence/presence of segmental glomerulosclerosis, T0/T1/T2 as tubular atrophy/interstitial fibrosis ≤ 25–50% > 50%, and C0/C1/C2 as absence/ < 25%/ ≥ 25% of crescent lesions.

The immunofluorescence samples were stained with fluorescein isothiocyanate (FITC)-conjugated antibodies specific for human IgG, IgM, IgA, C3, C4, and C1q (1:50, DAKO, Glostrup,Denmark). The degree of immunofluorescence was scored on a scale of 0–4 (score 0, negative; score 1, + ; score 2, +  + ; score 3, +  +  + ; score 4, +  +  + +).

### Immunofluorescence staining

Frozen tissues were embedded in OCT, cut into 5 μm sections, and then stored at – 20 ℃. Rabbit anti-human Mfn2 antibody (1:100; Cat No. M6319, Sigma-Aldrich), rabbit anti-human nephrin monoclonal antibody (1:100; ab50339, Abcam), rat anti-human collagen IV alpha 5 monoclonal antibody (1:100; C-452, Cosmo Bio), rabbit anti-human Parkin antibody(1:100; 14060-1-AP, Proteintech) were reacted with renal tissue at 4 °C overnight. AF488-conjugated donkey anti-rabbit IgG antibody (1:500; A-21206, Invitrogen), and FITC conjugated donkey anti-rat IgG antibody (1:500, A-18740, Invitrogen) were incubated for half an hour at 37 ℃. Sections were observed using a fluorescence microscope (Nikon 80i; Nikon, Tokyo, Japan).

### Electron microscopy

The renal biopsy specimens were fixed with osmic acid and glutaraldehyde, dehydrated, and embedded in EPON^™^ resin. Sections with a thickness of 1 mm were cut and stained with uranyl acetate and lead citrate. Thin sections were examined using a JEOL-1400 electron microscope (JEOL, Tokyo, Japan).

The degree of podocyte effacement was graded on a scale of 1–5 with 1 = podocyte effacement < 25%, 2 = 25–50%, 3 = 50–75%, 4 = 75–95%, and 5 =  ≥ 95%.

The number of mitochondria (M) and the area of podocytes (A) were assessed using ImageJ software. The ration M/A was used to evaluate the number of mitochondria per area and the length-to-width ratio of mitochondria was used to evaluate mitochondrial morphology. All pathological parameters were assessed and measured by two independent pathologists.

### Apoptosis assay

Tissue sections were stained with an In Situ Cell Death Detection Kit, Fluorescein, (Roche Cat No. 11684795910), and the slices were exposed to freshly prepared permeabilization solution for 2 min on ice (0.1% Triton X-100, 0.1% sodium citrate). After washing with PBS, the samples were resuspended in 50 µL of TUNEL (terminal deoxynucleotidyl transferase-mediated nick end-labeling (TUNEL) reaction mixture and incubated for 60 min in a dark, humidified environment. The samples were then washed with PBS, and the slides were examined using a fluorescence microscope (Nikon 80i; Nikon, Tokyo, Japan).

### Statistical analysis

Statistical analyses were performed using SPSS (version 23.0; SPSS Inc., Chicago, IL, USA) and GraphPad Prism (version 5.0; GraphPad Software, Inc., La Jolla, CA, USA). Fisher's exact test or the χ^2^ test was used to compare qualitative data, and the Wilcoxon rank-sum test was used for continuous variables. Spearman’s correlation test was used to assess the strength of the association between Mfn2 levels and clinical or pathological variables. Logistic regression analysis was performed to ascertain the relationship between Mfn2 expression and podocyte effacement. Statistical significance was set at* P* < 0.05.

## Results

### Patient characteristics at the time of renal biopsy

One hundred and fourteen patients who underwent renal biopsy between April 2022 and August 2022 were enrolled, including 57 males (50%) and 57 females (50%). The mean age was 39.4 ± 12.1 years. Thirty-eight patients had hypertension, but no diabetes was found at the time of renal biopsy. Sixty-four patients had stage 1 chronic kidney disease (CKD1), 26 had CKD2, 22 had CKD3, and only 2 had CKD4 [18].

### Mfn2 staining

**Fig. 1:**
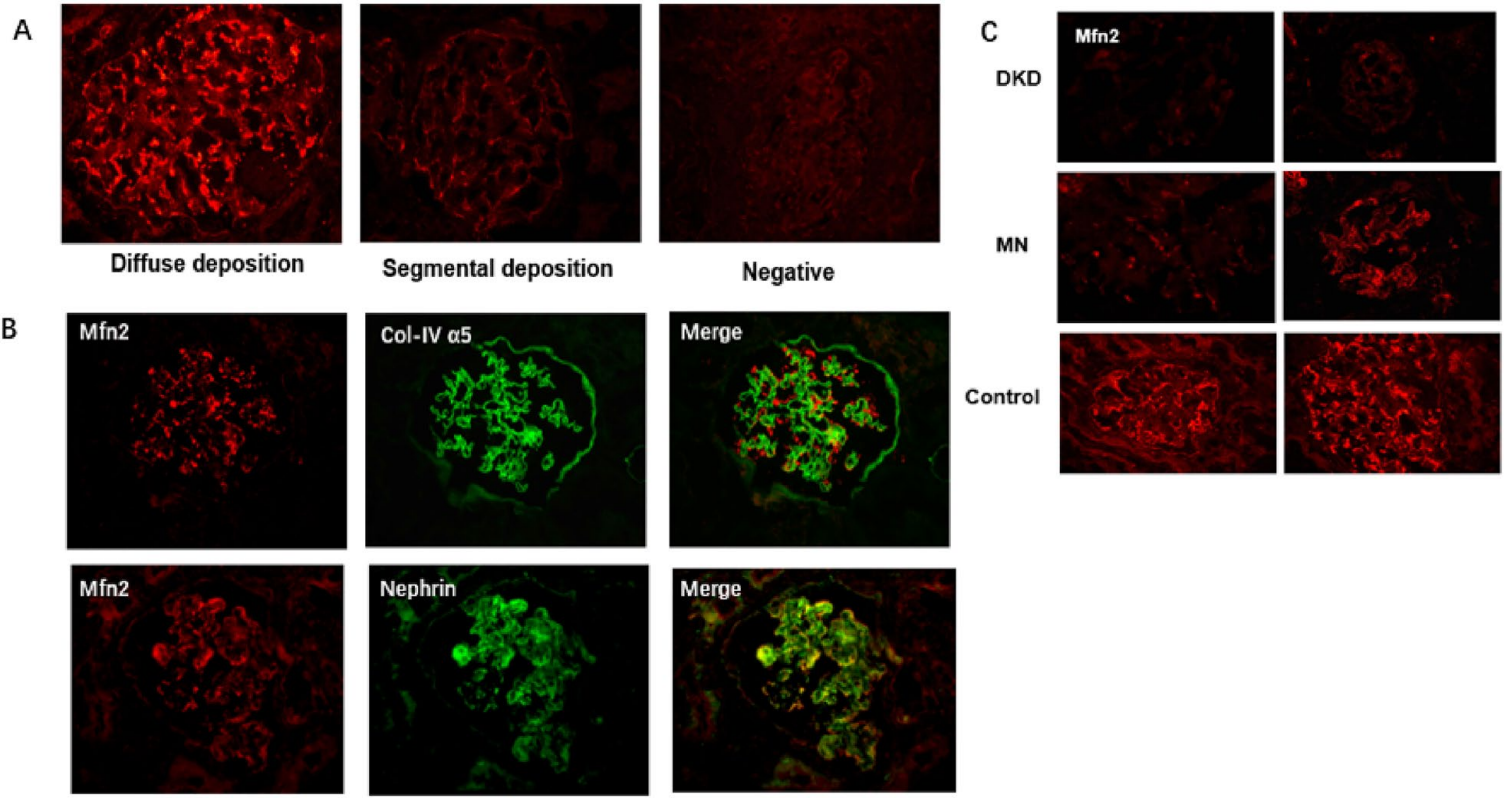
Mfn2 expression in patients with IgAN. **A** Representative images of immunohistochemical staining of Mfn2 in glomeruli per group (original magnification × 400). Mfn2 expressed globally or segmentally in the glomeruli were divided into positive group, no/trace Mfn2 staining in glomeruli was defined as negative group. **B** The location and form of Mfn2 expression in glomeruli. Mfn2 was co-stained with nephrin and Col-IV α5. Mfn2 deposition in two different types, granular deposition or deposition along the GBM at the same time (original magnification × 400). **C** Representative images of immunohistochemical staining of Mfn2 in DKD, MN and normal control (original magnification × 400)

Mfn2 was detected in glomeruli at different intensities, and a staining threshold was used to divide the patients into two groups. The positive group was defined as Mfn2 expressed globally or segmentally in the glomeruli, and the negative group was defined as no or only traces of Mfn2 staining in glomeruli (Fig. 1A).

The location of Mfn2 expression was determined by double-immunofluorescence staining with anti-Col IV α5 or anti-nephrin antibody. The results show that Mfn2 was located outside the glomerular basement membrane (GBM) and co-localized with nephrin, indicating that Mfn2 is expressed in podocytes (Fig. 1B). We also found that Mfn2 deposition occurred in two different ways: granular deposition or deposition along the GBM at the same time (Fig. 1B) We also detected Mfn2 expression in five DKD and five membranous nephropathy (MN) patients as disease controls, and five precancerous tissues from kidney tumors served as normal controls. As shown in Fig. 1C, in the normal control group, we detected diffuse Mfn2 deposition in the glomeruli. While all five of the DKD patients did not have detectable Mfn2 deposition. In MN, all five MN patients had segmental or traces of Mfn2 staining in glomeruli, and there were no significant differences in the deposition site and form of Mfn2 in MN compared with IgAN.

**Fig. 2:**
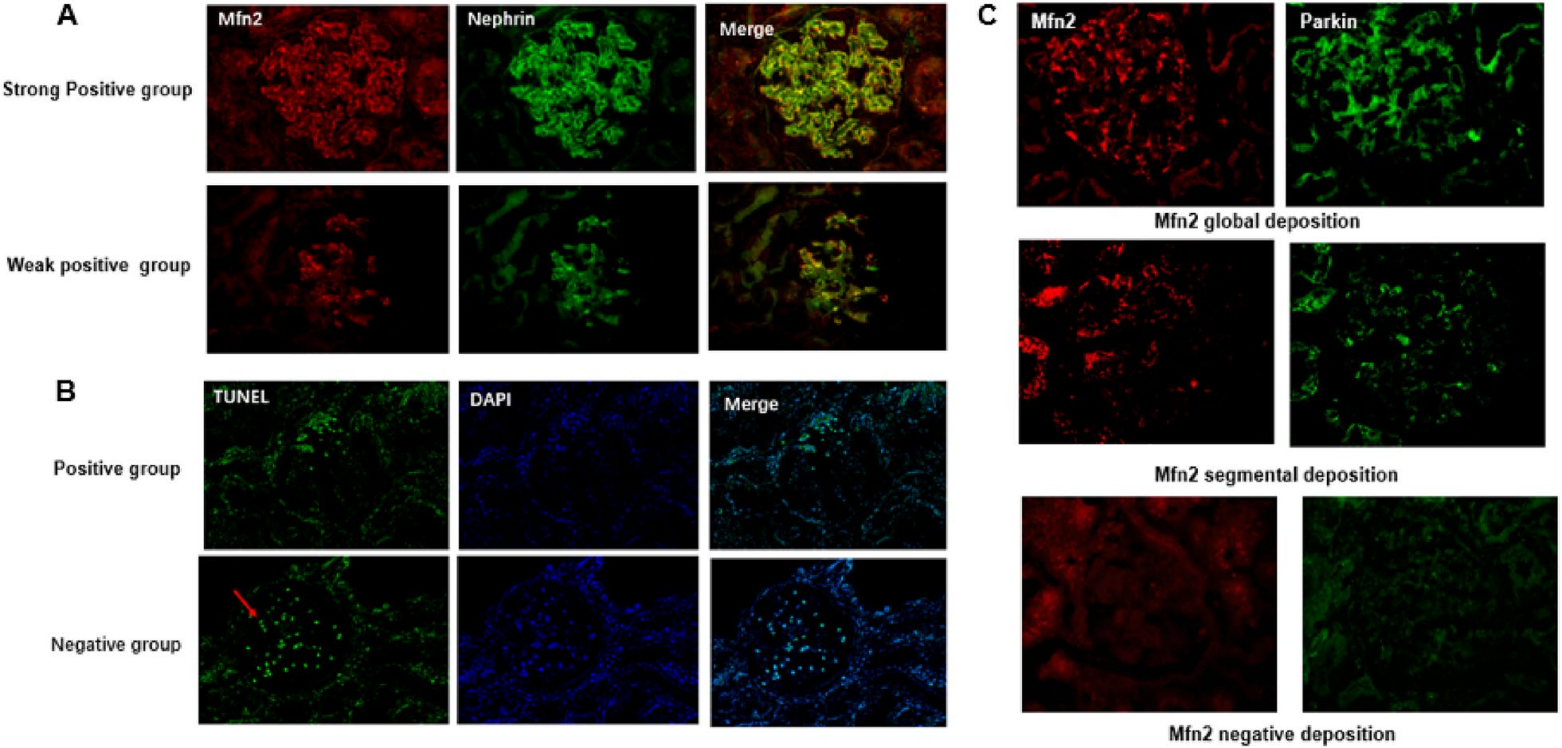
The association between Mfn2 expression and podocyte apoptosis and mitophagy. **A** Representative images of immunofluorescent staining of Mfn2 and nephrin in glomeruli per group (original magnification × 400). **B** Representative images of TUNEL staining in different group, Red arrows marked TUNEL positive cells (original magnification × 400). **C** Representative images of Mfn2 and Parkin staining in different group (original magnification × 400)

We further compared the correlation between Mfn2 and nephrin expression. The results show that the fluorescence intensity of Mfn2 decreased in parallel with the intensity of nephrin expression, indicating that there was probably a correlation between Mfn2 and nephrin expression (Fig. 2A).

Meanwhile, TUNEL staining showed that more positive cells were observed in the glomeruli in the Mfn2-negative group than in the positive group, indicating that podocytes without Mfn2 expression may be in the process of undergoing apoptosis (Fig. 2B).

### The correlation between Mfn2 expression and clinicopathological features

As shown in Table 1, 28 patients (24.56%) were assigned to the negative group, whereas 86 patients (75.44%) were assigned to the positive group. There were no significant differences in terms of age, gender, or blood pressure between the two groups, although the patients in the Mfn2-negative group had lower serum albumin (34.43 ± 4.64 g/L vs. 36.48 ± 3.52 g/L,* P* = 0.015) and eGFR levels (76.59 ± 35.38 mL/min vs. 92.13 ± 25.35 mL/min, *P* = 0.013) than the positive group. Additionally, the 24 h proteinuria level (2.48 ± 2.72 g/d vs. 1.27 ± 1.31 g/d, *P* = 0.002), Scr (107.39 ± 57.97 μmol/L vs. 84.70 ± 34.95 μmol/L, *P* = 0.015), and BUN (7.36 ± 4.45 mmol/L vs. 5.68 ± 2.14 mmol/L, *P* = 0.008) were much higher in the Mfn2-negative group than the positive group (shown in Table 1).

**Table 1:** Baseline data of patients with IgAN in the Mfn2-negative group and Mfn2-positive group

	Mfn2 ( +) (n = 86)	Mfn2(-) (n = 28)	*P* value
Diastolic blood pressure (mmHg)	79.30 ± 11.63	80.89 ± 14.06	0.552
Systolic blood pressure (mmHg)	125.95 ± 16.77	127.04 ± 13.30	0.757
Age (Year)	40.20 ± 11.71	37.00 ± 13.19	0.226
Sex(male/female)	44/42	13/15	0.663
Hypertension(%)	30 (34.88%)	9 (34.62%)	0.825
Scr (μmol/L)	84.70 ± 34.95	107.39 ± 57.97	0.015
BUN (mmol/L)	5.68 ± 2.14	7.36 ± 4.45	0.008
UA (μmol/L)	374.26 ± 112.15	389.46 ± 104.38	0.528
Alb (g/L)	36.48 ± 3.52	34.43 ± 4.64	0.015
24hPro (g/d)	1.27 ± 1.31	2.48 ± 2.72	0.002
GFR (mL/min)	92.13 ± 25.35	76.59 ± 35.38	0.013
Serum IgA	325.24 ± 106.58	303.82 ± 80.10	0.332

The renal pathological parameters showed higher S/T scores (92.86% vs. 70.93% and 42.85% vs. 15.12%, respectively, *P* < 0.05) and C1q deposits (46.4% vs. 25.6%, *P* = 0.05) in the Mfn2-negative group than in the positive group. Furthermore, a higher degree of podocyte effacement was observed in the negative group (2.78 ± 1.24 vs. 2.10 ± 0.81, *P* = 0.02) than in the positive group by electron microscopy (Table 2).

**Table 2:** Renal pathological parameters of patients with IgAN in the Mfn2-negative group and Mfn2-positive group

	Mfn2 ( +) (n = 86)	Mfn2(−) (n = 28)	P
M [n (%)]	M0 0 (0%) M1 86 (100%)	M0 1 (3.57) M1 26(96.43%)	0.25
E[n (%)]	E0 45 (52.32%) E1 41 (47.67%)	E0 15 (53.57%) E1 13 (46.43%)	0.58
S [n (%)]	S0 25 (29.07) S1 61 (70.93%)	S0 2 (7.14%) S1 26 (92.86%)	0.04
T [n (%)]	T0 73 (84.88%) T1 13 (15.12%) T2 0 (0%)	T0 14 (50%) T1 12 (42.85%) T2 2 (7.14%)	0.01
C [n (%)]	C0 38 (44.19%) C1 47 (54.65%) C2 1 (1.16%)	C0 10 (35.71%) C1 18 (64.29%) C2 0 (0%)	0.58
Intensity of IgA deposition	2.98 ± 0.32	2.89 ± 0.42	0.21
Intensity of IgG deposition	0.72 ± 0.65	0.71 ± 0.62	0.96
Intensity of IgM deposition	1.14 ± 0.33	1.37 ± 0.63	0.07
Intensity of C3 deposition	2.24 ± 0.59	2.21 ± 0.73	0.88
C4 deposition [n (%)]	6 (7.1%)	3 (10.7%)	0.68
C1q deposition [n (%)]	22 (25.6%)	13 (46.4%)	0.05
M/A	0.112 ± 0.483	0.156 ± 0.67	0.001
Length to width ratio	2.03 ± 0.93	1.23 ± 0.24	0.0001
Degree of podocyte fusion	2.10 ± 0.81	2.78 ± 1.24	0.02

**Fig. 3:**
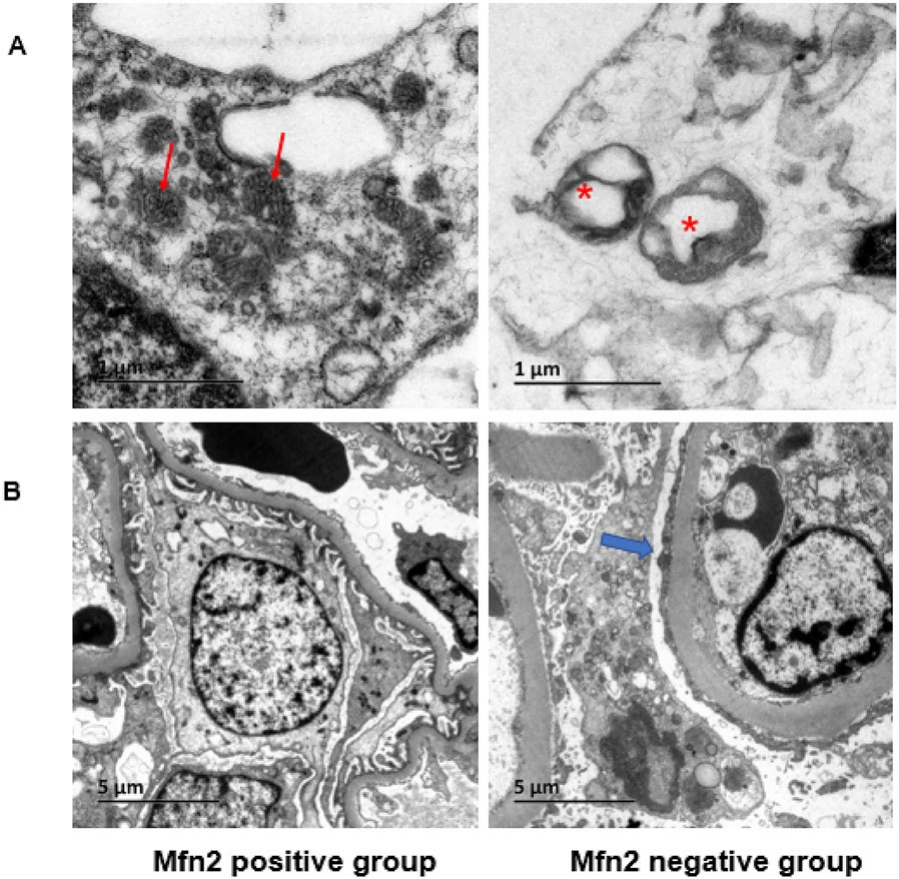
Mitochondria changes in patients with IgAN. The Transmission Electron Microscopy (TEM) images of Mitochondrial morphology and number in Mfn2 negative and positive group. (**A** The morphology of mitochondria in Mfn2 positive and negative group. The red arrows marked normal mitochondria Swollen and injured mitochondria marked by stars (original magnification × 20,000) **B** Representative images of mitochondria in podocyte with Mfn2 negative and positive group. In negative Mfn2 showed severe podocyte effacement marked by bule arrow (original magnification × 5000)

Accumulating evidence has revealed that PTEN-induced putative kinase 1 (PINK1) and Parkin participate in mitophagy during cardiac and kidney injury [14, 15]. PINK1-activated Parkin translocates to mitochondria with low membrane potential to initiate the autophagic degradation of damaged mitochondria [19] As a receptor of Parkin, Mfn2 is involved in the regulation of PINK1/Parkin-regulated mitophagy [14]. Therefore, we compared Parkin expression between the two groups, as shown in Fig. 2C. Parkin expression in glomeruli paralleled Mfn2 expression, with Parkin expression at the same time in the Mfn2-positive group, while there was no Parkin staining in the Mfn2-negative group. In addition to the detection of autophagy-related proteins, we also directly observed morphological and structural changes in mitochondria using TEM. Comparison of the number of mitochondria per unit area of podocyte (M/A) between the two groups showed that the M/A ratio was significantly higher in the Mfn2-negative group than in the positive group (0.156 ± 0.67 vs. 0.112 ± 0.483,* P* = 0.001), indicating that more mitochondria were present in the Mfn2-negative group than in the positive group (Table 2). The morphology of mitochondria was evaluated by comparing the length-to-width ratio, and the results showed that most of the mitochondria in the negative group exhibited a punctate or round shape with the disappearance of ridges, whereas they had an oval shape with normal ridges in the Mfn2-positive group (Fig. 3). Furthermore, we found that the length-to-width ratio of mitochondria was much lower in the Mfn2-negative group than that in the positive group, indicating that impaired mitochondria were common in the Mfn2-negative group (Table 2, Fig. 3).

### Correlation between Mfn2 quantitative fluorescence intensity and clinical data

**Fig. 4:**
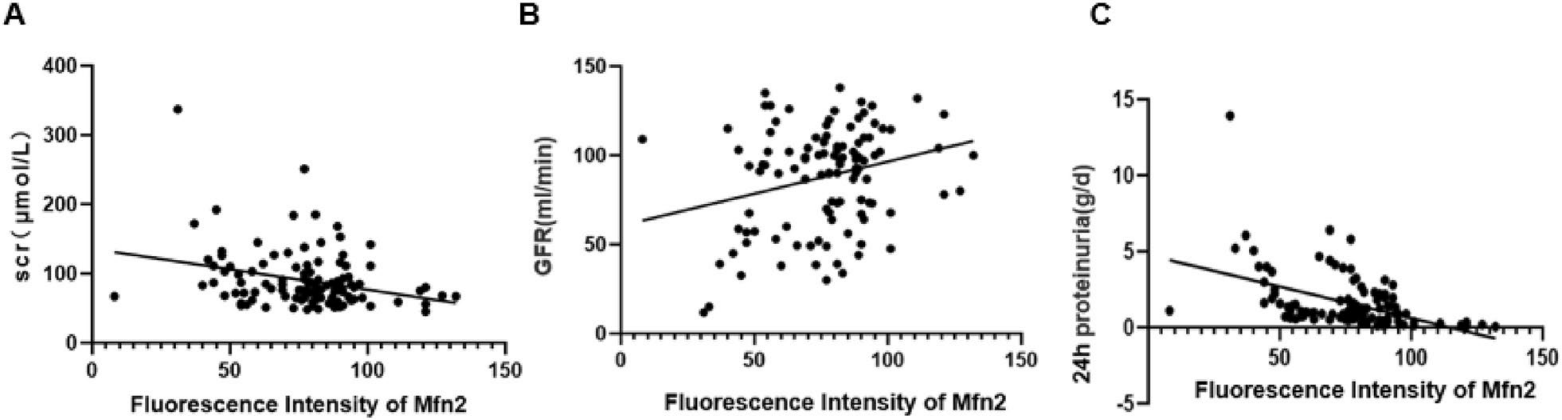
Line correlation between quantitative fluorescence intensity of Mfn2 and clinical data. Expression levels of Mfn2 in glomeruli negative correlated with Scr and proteinuria, positive correlated with GFR

The Mfn2 staining intensity in glomeruli was quantified using ImageJ software. The fluorescence intensity was 85.49 ± 16.05 in the Mfn2-positive group versus 50.57 ± 8.89 in the Mfn2-negative group, which was significantly different (*P* = 0.003). Correlation analysis showed that the intensity of Mfn2 was negatively correlated with the levels of Scr (r = − 0.232, *P* = 0.013), 24 h proteinuria (r = − 0.541, *P* = 0.001), and the degree of podocyte effacement (r = − 0.323,* P* = 0.001), whereas it was positively correlated with eGFR (r = 0.213,* P* = 0.025) (Fig. 4).

Logistic regression analysis showed that the Mfn2-negative group had a significantly higher risk of severe podocyte effacement (≥ 50%) than that of the positive group (OR = 3.061, *P* = 0.019), which suggests Mfn2 negativity indicates a higher possibility of podocyte injury.

## Discussion

In our study, we found that nearly 75.44% of patients with IgAN had Mfn2 expression in the kidney. Through immunofluorescence co-staining of Mfn2 with Col IV α5 and nephrin, we found that Mfn2 was mainly expressed in podocytes, and the intensity of Mfn2 decreased consistently with nephrin expression. TUNEL staining revealed an increase in podocyte apoptosis in the Mfn2-negative group. These findings indicate that Mfn2 expression is associated with podocyte injury in IgAN patients. In patients with DKD, Cao et al. similarly found a dramatic reduction in Mfn2 expression in DKD patients compared to the expression in healthy individuals, along with increased podocyte apoptosis, as detected by TUNEL and decreased synaptopodin expression [16].

As a mitochondrial membrane protein, Mfn2 participates in mitochondrial fusion and contributes to the maintenance and functioning of the mitochondrial network [20]. Additionally, Mfn2 is also involved in the regulation of mitophagy [13]. Mfn2 deletion has been reported to suppress mitophagy in mouse embryonic fibroblasts, cardiomyocytes, and macrophages. [13–15] Cao et al. reported that high glucose (HG)-induced podocyte mitochondrial dysfunction, MAMs reduction, and increased apoptosis in vitro were accompanied by downregulation of Mfn2 [15]. Jiang et al. found that palmitic acid (PA)-induced podocyte injury was accompanied by downregulation of Mfn2 and inhibition of mitophagy [21]. Our previous in vitro and animal model studies have indicated that overexpression of Mfn2 can inhibit puromycin aminonucleoside (PAN)-induced podocyte apoptosis. In the present study, we made similar findings: patients lacking Mfn2 expression had more serious podocyte injury and mitophagy. Electron microscopy showed that the degree of podocyte effacement was more pronounced in the Mfn2-negative group. By observing the number and the morphological structure of mitochondria in podocytes, we discovered that, compared with the positive group, the number of mitochondria per unit area was higher in the Mfn2-negative group, while the length-to-width ratio of mitochondria was smaller, indicating that there were more damaged mitochondria in podocytes in the Mfn2-negative group. Lack of Mfn2 inhibits mitophagy, which results in the accumulation of damaged mitochondria and excessive ROS production in cells, ultimately promoting podocyte apoptosis, which causes podocyte effacement and impairs the selective filtration of GBM, thereby leading to proteinuria.

Podocyte injury plays an important role in IgAN. Podocyte dedifferentiation is associated with IgAN [3, 22, 23]. Podocytopenia (loss of podocytes) has been reported to correlate with proteinuria and renal outcomes in IgAN patients [24, 25]. Moreover, mitochondria play a key role in maintaining the function and structure of podocytes. In our study, we found that 75% of IgAN were Mfn2-positive and 24% were negative. The negative patients had higher levels of proteinuria and Scr, higher S/T scores, and a higher degree of podocyte effacement and M/A value, indicating that Mfn2 expression negatively correlates with the severity of IgAN. Furthermore, TUNEL staining demonstrated that negative Mfn2 expression was associated with severe podocyte apoptosis, and the expression of Mfn2 was an independent risk factor for the severity of podocyte effacement, thus indicating that a reduced Mfn2 results in inhibition of mitophagy and the accumulation of damaged mitochondria and excessive ROS production in the cytoplasm. Ultimately, it promotes podocyte apoptosis and exacerbates IgAN progression.

## Conclusion

In IgAN, Mfn2 is mainly expressed in podocytes and is negatively correlated with proteinuria and renal function. A lack of Mfn2 in podocytes indicates severe podocyte injury and a high degree of podocyte effacement. Thus, Mfn2 could be a useful indicator of the severity and poor prognosis of IgAN.

This article still has several shortcomings. Firstly, it is a single-center study with a limited sample size. Additionally, due to a short-term follow-up, the treatment and outcome data have not been collected. Secondly, this article does not include a study of other mitochondrial morphology-related proteins such as mitofusin 1 (Mfn1), optic atrophy 1 (OPA1), and dynamin-related protein 1 (Drp1).

Further studies are necessary to enhance the understanding of the correlation between Mfn2 and the outcome in IgAN patients, as well as to investigate other mitochondrial fusion and fission proteins.

## Data Availability

The data sets used or analysed during the current study are available from the corresponding author on reasonable request.

## Abbreviations

Mfn2: Mitofusin2
IgAN: IgA nephropathy
eGFR: Estimated glomerular filtration rate
Scr: Serum creatinine
BUN: Blood urea nitrogen
ESRD: End stage renal diseases
OMM: Outer mitochondrial membrane
DKD: Diabetic kidney disease
MAMs: Mitochondria-associated ER membranes
Alb: Serum albumin
CKD: Chronic kidney disease
GBM: Glomerular basement membrane
HG: High glucose
PA: Palmitic acid
PAN: Puromycin aminonucleoside
FSGS: Focal segmental glomerulosclerosis
Gd-IgA1: Galactose-deficient IgA1
IgA1: Immunoglobulin A1
ER: Endoplasmic reticulum
ROS: Reactive oxygen species
